# The efficacy of an extraoral scavenging device on reduction of splatter contamination during dental aerosol generating procedures: an exploratory study

**DOI:** 10.1038/s41415-020-2112-7

**Published:** 2020-09-11

**Authors:** Shakeel Shahdad, Tulsi Patel, Annika Hindocha, Neil Cagney, Jens-Dominik Mueller, Noha Seoudi, Claire Morgan, Ahmed Din

**Affiliations:** 1 Honorary Clinical Professor in Oral Rehabilitation & Implantology and Consultant in Restorative Dentistry, Barts and The London School of Medicine and Dentistry, Queen Mary University of London, Barts Health NHS Trust, The Royal London Dental Hospital, London, UK; 2 Dental Core Trainee, Restorative Dentistry and General Duties, Barts Health NHS Trust, The Royal London Dental Hospital, London, UK; 3 Lecturer School of Engineering and Materials Science, Queen Mary University of London, Mile End Road, London, E1 4NS, UK; 4 Reader in Computational Fluid Dynamics and Optimisation, School of Engineering and Materials Science, Queen Mary University of London, Mile End Road, London, E1 4NS, UK; 5 Senior Clinical Lecturer in Oral Microbiology, Centre for Oral Immunobiology and Regenerative Medicine; Barts and the London School of Medicine and Dentistry, Queen Mary University of London, London, E1 2AD, UK; 6 Consultant in Restorative Dentistry, Barts Health NHS Trust, The Royal London Dental Hospital, London, UK; 7 Post-CCST Speciality Registrar in Orthodontics, Barts Health NHS Trust, The Royal London Dental Hospital, London, UK

## Abstract

**Introduction **This study was conducted in light of the SARS-CoV-2 pandemic, which brought UK dentistry to a standstill. The market has seen a recent influx of unproven extraoral scavengers (EOSs), which claim to reduce the risk of particulate spread.

**Aims **To investigate the efficacy of a commercially available EOS device on contamination reduction during dental aerosol generating procedures (AGPs). The secondary aim was to investigate differences between open and closed dental operatories.

**Method **Dental procedures were simulated on a dental manikin using citric acid (10%) added to the water lines with universal indicating paper (UIP) placed in strategic locations in the operatory, on the clinician and assistant. Chromatic change related to settling of splatter containing citric acid on the UIP was analysed to calculate percentage intensity of splatter contamination.

**Results **EOSs resulted in 20% reduction in frequency and 75% reduction in mean intensity of contamination of operatory sites. There was a 33% and 76% reduction in mean intensity contamination for clinician and assistant, respectively. Use of rubber dam and four-handed dentistry resulted in further reduction.

**Discussion **This exploratory study demonstrates contamination by splatter in a simulated dental setting. The concern in dentistry regarding aerosol requires further quantitative investigation of smaller particles.

**Conclusions **The routine use of four-handed dentistry and rubber dam should continue where possible to maximise risk mitigation during AGPs. However, on the basis of our findings, the use of an EOS device can further mitigate the magnitude and concentration of splatter.

## Key points

- The extraoral scavenging device resulted in 20% reduction in frequency and 75% reduction in mean intensity contamination of operatory sites.
- Four-handed dentistry, rubber dam and extraoral scavenger used in conjunction reduce contamination.
- Open clinics are no worse than closed surgeries for conducting aerosol generating procedures in this setting.

## Introduction

In the midst of the SARS-CoV-2 pandemic, increasing emphasis has been placed on limiting the spread of the virus and protecting healthcare workers and the public. Clinical dentistry poses an exposure risk to dental professionals and patients, largely owing to the nature of dental procedures which often generate airborne particulates contaminated with bacteria, blood, viruses and fungi.^1^^,^^2^ From 25 March 2020 to 8 June 2020, all dental practices in the UK were closed for routine care.^3^^,^^4^ With the reopening of practices, concerned dental professionals have been seeking strategies to minimise the risk of spread and contamination from SARS-CoV-2. There is debate as to whether SARS-CoV-2 is transmissible via an airborne route, with related evidence being contentious and incomplete.^5^^,^^6^^,^^7^ The most comprehensive scientific evidence available however, associates procedures capable of generating an aerosol with having an increased risk of SARS-CoV-2 transmission.^6^^,^^8^

Within dentistry, the dissemination of microbes from the patient's mouth to the clinician can occur in three possible ways: direct contact with contaminated droplets; indirect contact with contaminated surfaces or instruments; and close-range aerosol transmission during dental aerosol generating procedures (AGPs).^1^ To protect the dental team and patients from infection transmission, a comprehensive infection prevention and control protocol is recommended, including hand hygiene, instrument and hard-surface decontamination, use of appropriate personal protective equipment (PPE), triaging and risk assessment, and proper ventilation. Moreover, baseline screening is also advocated and a risk reduction strategy to reduce aerosol from AGPs should be instituted as follows:

1. High-volume suction (HVS)
2. Proper isolation
3. Use of pre-procedural antiseptic mouthrinse.^9^

HVS considerably reduces operating site contamination.^10^^,^^11^^,^^12^^,^^13^ In addition, rubber dam has been shown to further reduce potential airborne contamination by isolating individual teeth.^7^ However, rubber dam is not universally applicable to all dental procedures; for example, periodontal, oral surgery and orthodontic procedures.

Since the beginning of the pandemic, there has been an increase in availability of extraoral scavenger (EOS) devices on the market. Previous studies have been limited to evaluating the effectiveness of HVS in reducing airborne particulates^10^^,^^11^^,^^12^^,^^13^ and there is a paucity of evidence supporting routine use of EOS devices.^14^^,^^15^ Previous studies have utilised experimental rather than purpose-manufactured equipment for extraoral airborne particulate scavenging.

The primary aim of this exploratory study was to investigate the efficacy of a commercially available EOS device for routine dental procedures as an adjunct to reduce splatter contamination. The secondary aim was to investigate any differences between splatter contamination in an open clinic compared to a closed surgery within a dental hospital setting.

## Methods and materials

This *in vitro* experimental study was conducted at The Royal London Dental Hospital, UK, to investigate the efficacy of an EOS device in reducing splatter generated during various dental procedures (Appendix 1, 2 and 3). Experiments were predominantly conducted in a closed surgery (floor surface 16.8 m^2^), with some procedures replicated in an open, multi-chair clinic (single bay floor surface 10.0 m^2^). The centralised air exchange system (high-efficiency particulate air [HEPA] filtered) remained functional during all procedures, with six air changes per hour.

**Fig. 1:**
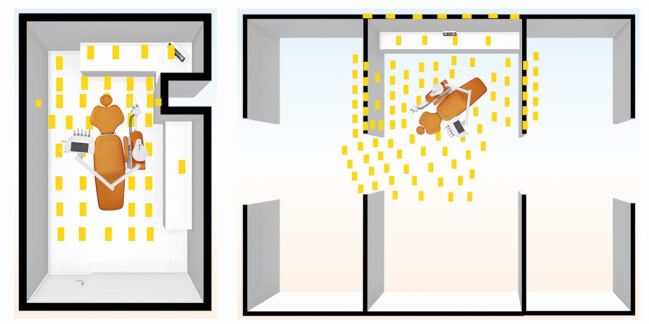
Diagrammatic representation of the closed surgery (left) and open clinic (right). Yellow boxes denote UIP locations. Additional UIP positions included: patient, bracket table, light, chair-mounted screen and ceiling

**Fig. 2:**
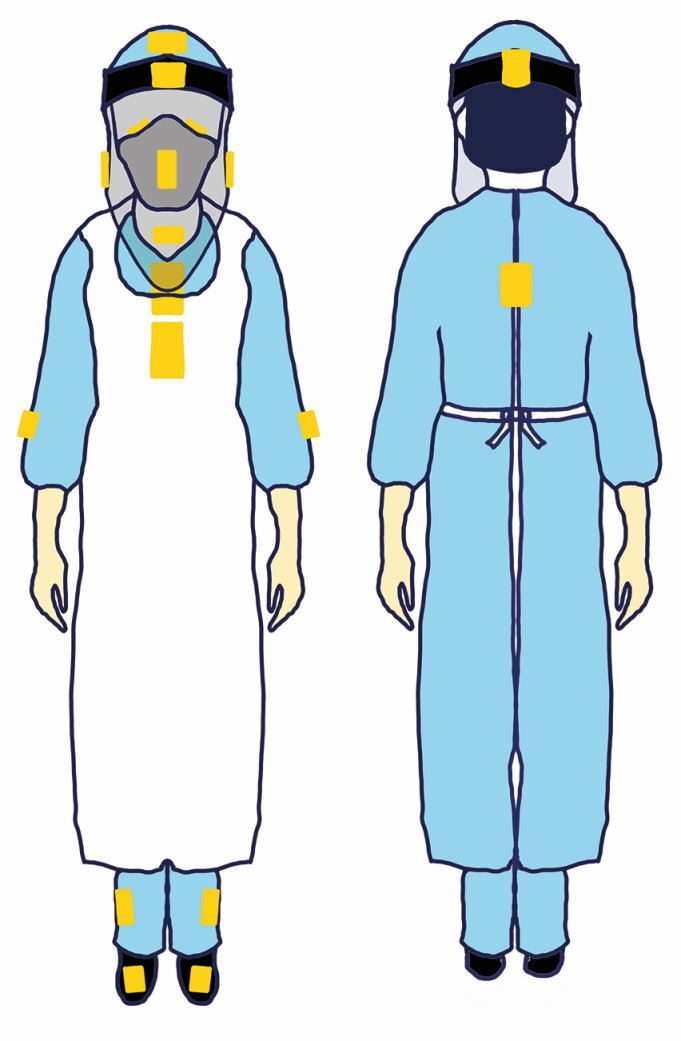
Diagrammatical representation of UIP on the clinician and assistant. Additional UIP was placed on the inside of the visor and on the mask

A dental manikin with thermoplastic teeth was set up on a dental chair to simulate the patient to avoid unnecessary risks to operators. Universal indicator paper (UIP) (Johnson Test Papers Ltd, Oldbury, UK) was strategically placed at fixed labelled sites within the surgery (Fig. 1) and on various parts of the clinician and assistant (Fig. 2). All distances were measured horizontally from the incisal edge of the maxillary left central incisor (21) to the centre of each UIP.

Citric acid (Intra Laboratories Ltd, Plymouth, UK) solution (10%) was placed in the water line of the dental chair. UIP has a sensitivity range of pH 1-14, which chromatically changed to red on contact with citric acid solution, therefore visually indicating contamination.

Air turbine (W&H Synea Turbine TA98LED Bürmoos, Austria) procedures were carried out with standard diamond burs and operated at full speed (360,000 rpm) with irrigation from the water line.

Full-mouth supragingival scaling was simulated with an ultrasonic scaler (Dentsply Cavitron Select SPS USA) at a maximum frequency (30 KHz) with water supplied from the dental chair.

The HVS (bore diameter 8 mm) and a standard saliva ejector (SE) were used during relevant procedures. When an assistant was present, the HVS and SE were operated using a typical four-handed dentistry technique. When only the SE was used (replicating procedures undertaken with no assistant), it was orientated and positioned contralaterally at the back of the oral cavity; with such procedures, the assistant was not present in the operatory.

**Fig. 3:**
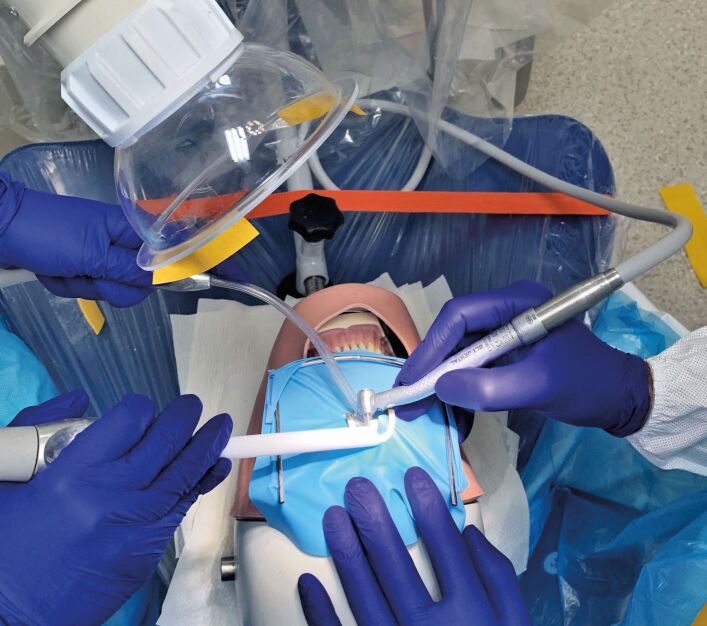
Photograph showing procedure setup with location of EOS in 5 o'clock position above the dental manikin at 0.15 m distance

The EOS unit (TM10, TopMed Dental Lighting Co. Ltd., Foshan, China) was used at maximum flow capacity (manufacturer specification of 310 m^3^/h) throughout the relevant procedures and for 20 minutes post-procedure. The EOS intake was consistently placed in the 5 o'clock position, 15-20 cm from the oral cavity (Fig. 3) with the head located between the patient and assistant. During procedures not involving the EOS, the unit was removed from the surgery.

Procedures were generally undertaken using the Barts and QMUL visor (Barts Health NHS Trust, London, UK; available from https://forms.gle/1wL5HbjTJ1StPGR88) as part of the enhanced PPE, which has an increased length of 420 mm and a width of 297 mm. In those procedures where a short visor was used, a disposable visor with a length of 220 mm and width of 330 mm was worn (Face Shield, Weihai Dishang Medical Technology Co. Ltd, Shandong Province, China).

The door of the closed surgery was kept shut during and after all procedures. After completion of each procedure, clinicians left the room for 20 minutes, allowing particles to settle. Visual examination of the UIP under bright operatory lights was undertaken by three research assistants verifying contamination; any UIP with red conversion was removed and its location logged for analysis.

**Fig. 4:**
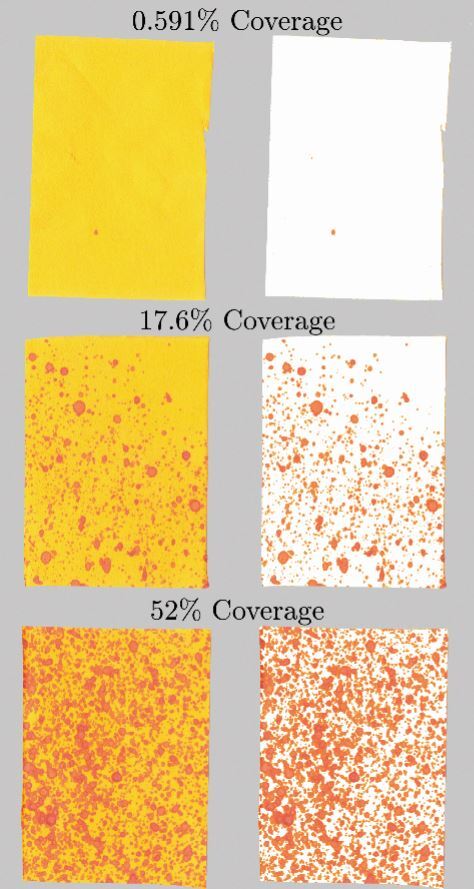
Example image analysis reporting intensity of contamination (%) by colour threshold analysis for three different sites. Left indicates the raw image with the right indicating the contaminated colour extraction

Contaminated UIPs were scanned (HP Colour Laser Jet Managed MFP E87640, Boeblingen, Germany) at 600 dpi. The images were then imported for image analysis (Matlab R2020a, The MathWorks Inc. USA) and analysed using thresholding to decompose each UIP into red contaminated and yellow uncontaminated regions (Fig. 4). Thresholding was repeated for selected strips, ensuring the results were not affected by choice of threshold value. Visual inspection was conducted to confirm the software correctly identified all visible contaminated regions.

Descriptive data comparing the spread of the citric acid solution were tabulated, allowing for the range (distance from patient) of spread to be assessed. Any contamination (including a single point) was recorded as a contaminated site and analysed. The results were reported differentiating the open clinic and closed surgery, with the operatory denoting the physical room components, and the clinician and assistant reported separately. Image analysis produced a percentage (%) coverage of UIP. Results were reported in terms of the maximum intensity (highest percentage coverage of a single UIP), the mean intensity (average coverage of all contaminated UIPs) and frequency (n = number of contaminated UIPs).

## Results

There was a considerable degree of variability of contamination produced by different procedures and between open clinic and closed surgeries (Appendices 1, 2 and 3).

### EOS

In general, when pooled for all procedures, EOSs reduced the mean intensity of contamination by 75% for the operatory sites, 33% for the clinician and 76% for the assistant (Table 1). Frequency was reduced by 20% in the operatory sites but remained unchanged for other sites.

**Table 1:** Percentage mean difference in frequency and intensity of contamination between procedures with and without EOS. Results reported for operatory, clinician and assistant sites separately

Result for each site	Without EOS	With EOS	Difference	Reduction (%)
Mean frequency of operatory sites contaminated (n)	5	4	1	20%
Mean percentage intensity of operatory contamination	2.9	0.72	2.18	75%
Mean frequency of clinician sites contaminated (n)	4	4	0	0%
Mean percentage intensity of clinician contamination	0.81	0.54	0.27	33%
Mean frequency of assistant sites contaminated (n)	2	2	0	0%
Mean percentage intensity of assistant contamination	1.01	0.24	0.77	76%

**Fig. 5:**
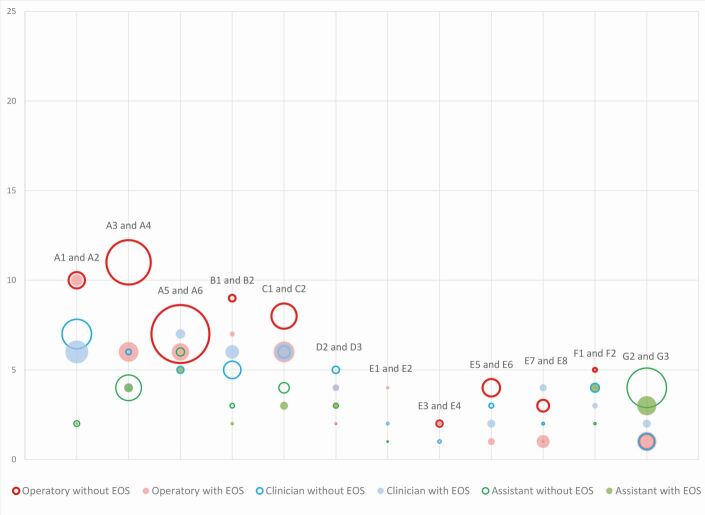
Scatter graph showing frequency (y-axis) and percentage intensity (data point size) of contamination, comparing equal procedures with and without EOS (inclusion of closed surgery and open clinic). Data labels represent procedure code. Note that A4 data point for frequency and mean intensity of contamination on clinician and assistant overlap

For air turbine procedures carried out in the closed surgery (A1 and A2), there was a reduction in mean intensity when using EOS devices. When the same procedure was repeated in an open clinic (A3 and A4), the operatory showed a reduction in both the frequency and the mean intensity (Fig. 5, Appendix 1). The procedure was again repeated with short visors in an open clinic (A5 and A6), which showed comparable trends with reduction of frequency and mean intensity.

When analysing the clinician, the contamination reduced in both frequency and mean intensity for the closed surgery when using EOSs (A1 and A2). There was a decrease in the frequency of contamination for the open clinic but an increase in the mean intensity (A3 and A4). When using short visors (A5 and A6), there was in increase in both the frequency and the mean intensity, with maximum intensity recorded on the chest.

On the assistant, there was no difference in mean frequency of contamination in the closed surgery or open clinic, although a decrease in mean intensity of contamination was noted with EOSs (A1 and A2; A3 and A4) (Appendix 2). The introduction of short visors on the open clinic led to a decrease in mean frequency and intensity when an EOS was used (A5 and A6), with the chest being the worst affected area.

Ultrasonic scaling procedures (E3-E8) were associated with a lower frequency and mean intensity of splatter than that observed with air turbine procedures (Fig. 5, Appendix 2). When using an EOS with an ultrasonic scaler, there was a reduction in mean intensity for all procedures, except during lower frequency ultrasonic use (E1 and E2).

In the open clinic (E7 and E8), ultrasonic contamination was lower in mean intensity compared to the closed surgery (E3 and E6) (Appendix 2). When the ultrasonic was used alone with SE (E5), there was greater frequency and intensity of contamination of the operatory. Adjunctive use of HVS (E3) and EOS (E4 and E6) resulted in further reduction in mean intensity. Contamination of the clinician followed a similar trend to that of the operatory, with small reductions with an EOS and four-handed dentistry (E3, E6, E7, E8) (Appendix 2). There was both frequency and maximum intensity of spread of zero on the assistant in four out of five procedures.

Lower frequency and mean intensity of contamination of the operatory (Fig. 5, Appendix 3) when compared to air turbine procedures was found with surgical sectioning of lower molars (G2 and G3). There was a reduction in mean intensity of contamination of the clinician and assistant when using an EOS (G2 and G3). The assistant was more contaminated than the clinician during all surgical procedures including implant placements (G1-J2).

The use of rubber dam and an EOS resulted in a reduction of mean and maximum intensity on the operatory, clinician and assistant in all procedures (B1, B2, D2 and D3), although an increase in operatory contamination was recorded for D3.

### Closed surgery

**Fig. 6:**
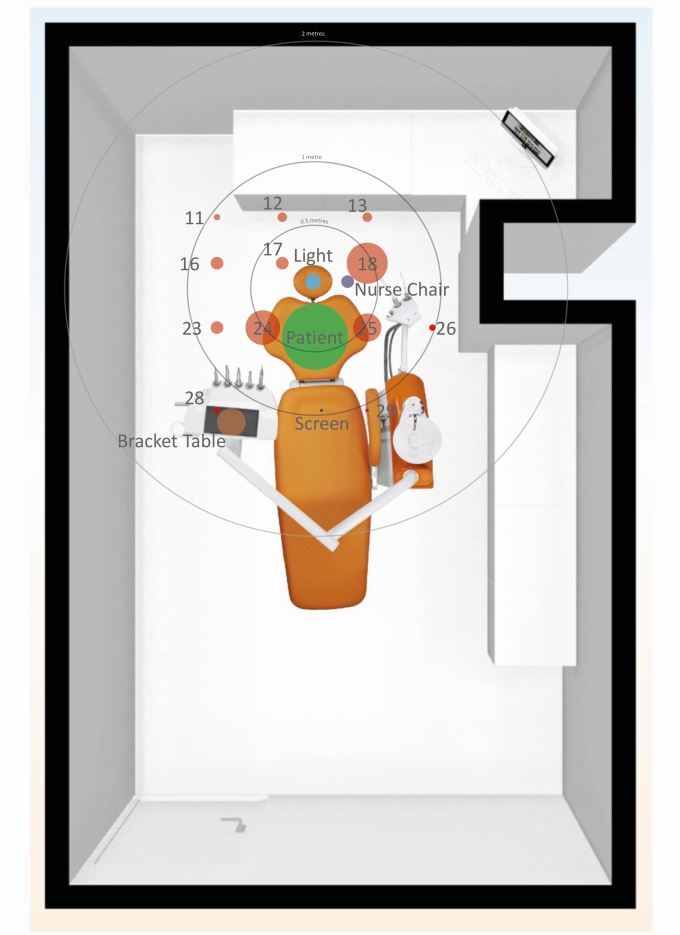
Closed surgery distribution of splatter contamination on UIP with labels representing site numbers. Size of data point indicates the frequency that site was contaminated across all closed surgery procedures. The UIP position is represented as green on the patient; red on the floor; navy blue on the assistant's chair; and orange on the bracket table

**Fig. 7:**
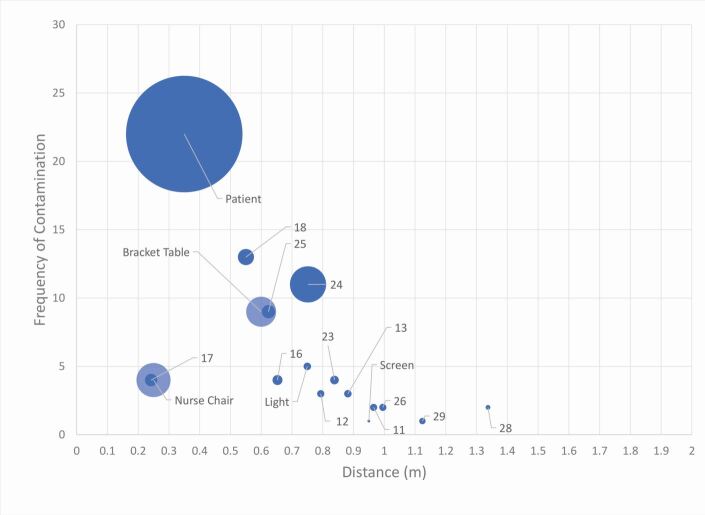
Frequency (y-axis) against distance (x-axis), with mean percentage coverage (%) represented by size of data point in a closed surgery. Labels represent the site numbers

The distribution in a closed surgery showed an increased concentration to within 1 m (Figures 6 and 7). The furthest site recorded was at 1.34 m in the 8 o'clock position (site 28).

The patient represented the most contaminated site (n = 22, at a distance of 0.35 m), followed by the 3 o'clock position (site 18; n = 13). The bracket table was contaminated with a mean intensity of 1.99% (0.6 m) and the assistant's chair with intensity of 2.58% (0.24 m).

There was no contamination on the adjacent walls (behind or to the left and right of the patient) or the ceiling. There was contamination on the chair-mounted display screen following one procedure (C4). Surprisingly, minimal contamination on the overhead operating light was recorded (n = 5; 0.12%) despite its close proximity to the patient (0.75 m).

### Open clinic

**Fig. 8:**
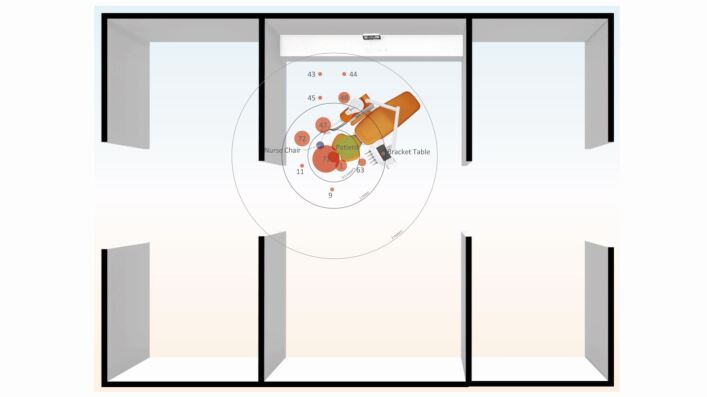
Open clinic distribution of splatter contamination on the UIP with labels representing site numbers. Size of data point indicates the frequency that site was contaminated across all open clinic procedures. The UIP position is represented as green on the patient; red on the floor; navy blue on the assistant's chair; and orange on the bracket table

**Fig. 9:**
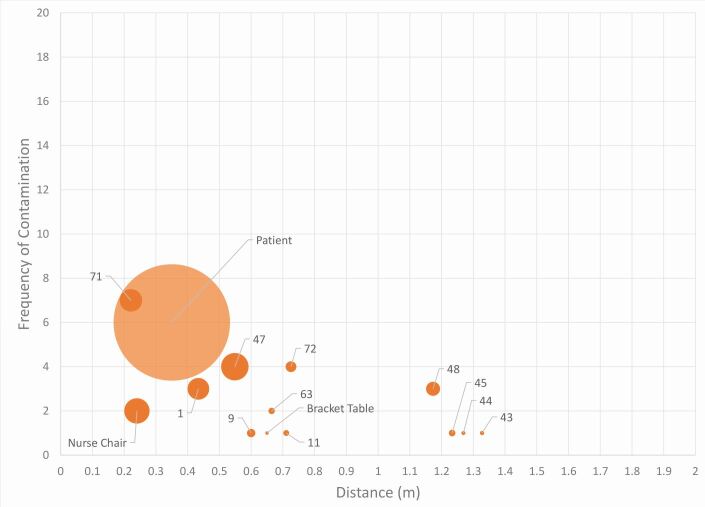
Frequency (y-axis) against distance (x-axis) with mean percentage coverage (%) represented by size of data point in an open clinic. Labels represent the site numbers

The distribution in open clinic was mostly within 1 m (Figures 8 and 9). Most contamination was concentrated close to the patient's head in a 1 o'clock position at site 71 (n = 7; 0.22 m). The furthest distance recorded was at 1.33 m in the 4 o'clock position (site 43).

The frequency of contamination was less in the open clinic compared to the closed surgery; the frequency and mean intensity of contamination on the patient was relatively low (n = 6; 29.4%) compared with the closed surgery (n = 22; 30.2%). Contamination of the bracket table was less frequent (n = 1; 0.02%) when compared to the closed surgery (n = 9; 1.99%).

There was no contamination above a height of 1.33 m and there was no contamination on top of the partition walls and beyond the immediate vicinity of the open clinic in any direction. There was also no recorded contamination on the overhead operating light, chair-mounted display screen, ceiling or adjacent walls.

### Clinician and assistant

**Fig. 10:**
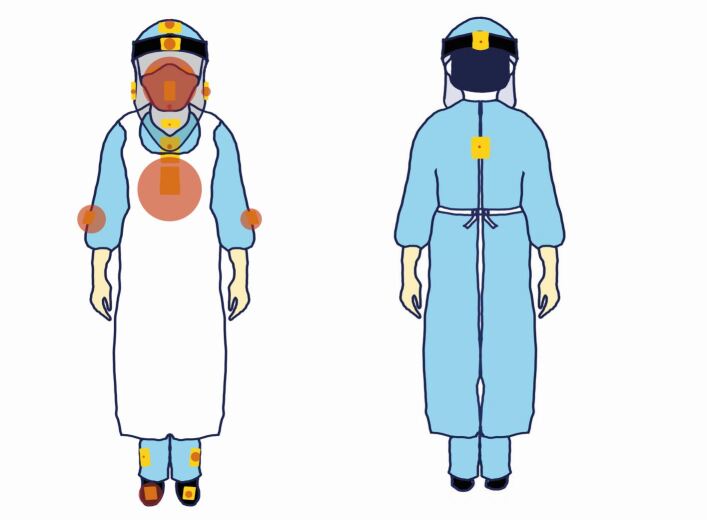
Graphic representation of frequency of contamination (data point size) on the operator (dentist) sites across all procedures

The clinician's chest was the most commonly contaminated site (n = 27 at an average distance of 0.3 m), showing a mean intensity of 3.72% (Fig. 10), with a maximum intensity of 24.52% during surgical sectioning of the 37 and 38 (G2) compared to any other procedure.

The front of the visor was the next most common site (n = 23) at an average distance of 0.32 m with a mean intensity of 4.09%. The maximum intensity (51.94%) was recorded during procedure A1.

For the assistant, the front of the visor was the most commonly contaminated site (Fig. 10) (n = 21) at 0.32 m with a mean intensity of 3.90% (A3). The maximum intensity, however, was recorded during surgical sectioning (G2; 71.07%).

### Surgical procedures

**Fig. 11:**
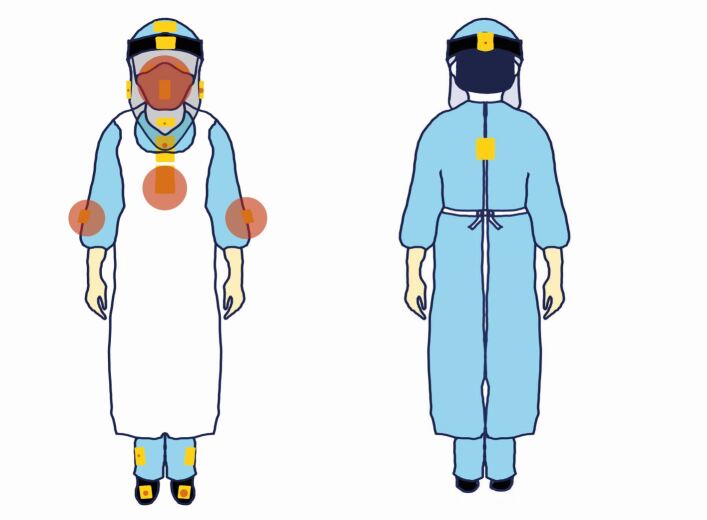
Graphic representation of frequency of contamination (data point size) on the assistant sites across all procedures

If surgical procedures are analysed, in isolation, there is a different trend between clinician and assistant. The assistant's chest (n = 7; 36.86%) and left forearm (n = 7; 2.26%) were the most contaminated; the maximum intensity recorded on the chest was 71.07% during surgical sectioning of the 37 and 38 (G2) (Fig. 11, Appendix 3). More contaminated sites were recorded on the assistant (nine) compared to the clinician (three).

When both clinician and assistant were reviewed closely, the lower half of the chest (n = 27) was more contaminated than the upper half (n = 2). There was no contamination seen on the inside of the visor.

### EOS validation

**Fig. 12:**
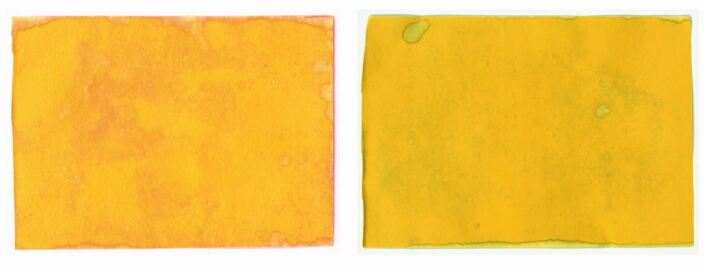
UIP confirmation of citric acid presence (red) on top surface of first filter (left) and underside of first filter (right) showing no citric acid. Due to the need for rehydration in this assessment, the right image shows the alkaline nature of water

On examination of the internal filtration components of the EOS, citric acid was only identified on the top surface of the first filter (out of five filter layers in total); there was no contamination through the thickness of this layer (Fig. 12).

## Discussion

Respiratory pathogens, including SARS-CoV-2, can colonise the oropharynx where the oral biofilm acts as a reservoir.^16^ Routine dental procedures such as drilling, scaling and polishing have the potential to aerosolise saliva and blood, causing airborne contamination.^17^^,^^18^ These particles can be absorbed across the respiratory mucosa and conjunctiva and penetrate the lungs, which can result in airborne transmission of SARS-CoV-2.^5^

Aerosol particles are smaller than 50 μm in diameter and remain airborne for prolonged periods.^1^^,^^10^ In contrast, splatter consists of a mixture of air, water or solid matter greater than 50 μm in diameter and can behave in a ballistic nature.^9^ Ballistic particles are discharged forcibly from the operating site and arc in a parabolic trajectory until they contact a surface.^9^

The spread and intensity of splatter and droplet creation, in isolation, were evaluated in the present study. Both are accepted to potentially harbour SARS-CoV-2 and were therefore used as a means of assessing the efficacy of the EOS device. We have, not evaluated aerosol generation in this study. Considerable variation in exposure levels of the clinician, assistant and patient were observed, influenced by the type of procedure, use of intraoral suction and EOS device. The most commonly affected areas included the chest, visor front, forearms and feet, with the clinician and assistant receiving varying levels of contamination depending on the procedure. The use of an elongated visor provided greater protection to the face and neck of the clinician and assistant from splatter, as no contamination of the internal visor surfaces or the upper chest behind the visor was noted throughout this study. The elongated visors protected the eyes, nose, mask and neck, all areas prone to exposure from splatter.^19^ Consequentially, the use of elongated visors, aprons, surgical gowns and easy-to-wipe, hole-free footwear is recommended by the authors.

This study confirmed the efficacy of HVS in reducing splatter contamination to dental personnel and patients, as seen in previous studies reporting significant reduction in splatter when large-bore HVS is utilised during AGPs.^9^^,^^13^^,^^20^^,^^21^^,^^22^

Previously, two different experimental EOS systems provided evidence for improved scavenging during AGPs and efficient prevention of air contamination with significantly lower bacterial count.^14^^,^^15^ While there were some procedures that showed an increase in operatory splatter contamination with the use of the EOS, there was a reciprocal decrease in clinician and assistant contamination. This could be attributed to the scavenging action of the EOS changing the airflow dynamics in the immediate area, thereby reducing exposure of the clinician and assistant. The current findings suggest that, in light of the SARS-CoV-2 pandemic, the four-handed approach to all dental procedures would enhance protection during AGPs.

The study demonstrated that the spread and intensity of contamination decreased with HVS and that the addition of the EOS device reduced this further. Notwithstanding, it is imperative that, for safety, such EOS devices have an effective filtration mechanism. This was validated in this study, with contamination limited to the top surface of the first filter only.

Ultrasonic scalers primarily debride via 'cavitation', resulting in the production of high pressures, which aids the cleaning process.^23^ This study showed ultrasonic scalers produce less splatter when compared to air turbines; however, this observation cannot be extrapolated to aerosol generation. HVS is known to be effective at decreasing the risk of disease transmission by reducing the number of microorganisms generated during ultrasonic scaling,^24^ and EOS devices have been shown to reduce blood-contaminated aerosols suspended in the air at 0.5 m and 1 m from the mouth.^11^ With regards to splatter generation, our results are in keeping with previous findings that the use of SEs alone during ultrasonic scaling results in more contamination by splatter than HVS.^11^^,^^21^

Furthermore, the reduction of the ultrasonic speed to 70% reduced the contamination of the operatory, clinician and assistant compared to 100% speed. It is therefore conceivable that four-handed dentistry for all procedures involving ultrasonic scalers set at 70% speed would likely minimise exposure.

The results established that rubber dam use led to a decrease in the number of sites contaminated and, more significantly, the intensity of contamination with and without the EOS. As such, the use of a rubber dam is recommended where possible to limit the source of contamination to the isolated tooth and reduce the resultant splatter production.^19^

On the basis of a previous study involving the use of UIP, a safe distance of six feet (1.83 m) surrounding the dental chair for personnel safety was recommended.^2^ Within the open clinic setting, the maximum recorded distance of splatter contamination was 1.33 m from the operating site, with the majority of the contamination found in the immediate vicinity of the patient. This evidence may imply that open clinic settings most commonly found within dental hospitals may be 'safer' than previously assumed. However, robust research and evidence concerning the dispersion of fine-particle aerosol is required to support this contention.

The limitations of this exploratory study are recognised; namely splatter in isolation has been evaluated at this stage with aerosol detection not undertaken. Furthermore, the colour of the unaffected UIP potentially contained varied elements of red, which may or may not have been picked up during analysis. Due to time constraints, the majority of the experiments were not repeated, and as a result, there may be anomalies in the results. Repeat experiments would have allowed for more robust statistical analyses allowing for outliers to be identified; however, repeats would still measure the surrogate marker (splatter). Moreover, identical experimental conditions were used throughout, with each experiment therefore acting as a positive control. The experiment was ultimately a simulation, eliminating patient factors such as the saliva, soft tissues and patient compliance, which could influence the efficacy of scavenging systems. Saliva, in particular, is the primary of these concerns, as it would act as a pathogenic reservoir and likely affect the outcomes.

It is recognised that clinicians and assistants will assume different positions for different procedures under different operating conditions. Within this study, all procedures were carried out by a right-handed clinician, potentially affecting the distribution of splatter; however, the quantity is likely to be similar, irrespective of handedness. It is also noted that the height at which the patient was reclined may impact upon the distance the particles travelled due to their parabolic nature. Nevertheless, the simulation was carried out using the most common patient position and procedures. In addition, clinical experience of the clinician may reduce operating times and therefore aerosol exposure.

The results indicate that the assistant was more affected in both frequency and intensity during surgical procedures than the clinician (Appendix 3). The straight surgical handpiece generated a larger volume of splatter, which did not show as individual splatter marks but rather a large volume exposure on the assistant's chest and, to a lesser degree, on the front of the visor. Due to the unusual pattern of splatter exposure, the procedure was repeated on the contralateral side. Nevertheless, the assistant still received the majority of the contamination. HVS was not used during surgical procedures as it would be considered perilous with open flaps. A surgical suction tip was used, which would reduce the suction efficiency, resulting in an inability to evacuate the irrigant efficiently during surgery. This procedure was the only simulation where the contamination with the use of the EOS device was slightly higher. This could be an experimental anomaly or could be due to the reduced efficacy of the EOS device extracting larger splatter particles. During the air turbine procedures, the removal of a stream of mist into the EOS device was visible to the naked eye.

Implant osteotomy preparation in the 36 and 46 region was carried with the handpiece set at 800 rpm. The amount of splatter generated was minimal and the use of an EOS did not seem to offer a substantial benefit, at least when considering contamination as a result of splatter. During the surgery, it was noticed that the positioning of the irrigation clip on the head of the handpiece was critical in determining the extent of the splatter generation. When ideally placed to deliver saline at the tip of the implant drill, the splatter generated was minimal. However, if the clip rotated even slightly, the splatter generated was spread over a much larger area and consequently resulted in more splatter generation, which may become contaminated with aerosolised blood, saliva and bone in the clinical setting. Therefore, the authors recommend that all procedures requiring the use of a fast or surgical handpiece should be considered AGPs, until objective measurements of aerosol generated by these various procedures are available.

## Conclusions

Within the limitations of this simulated exploratory study, an EOS device can tentatively be recommended for reduction in contamination by splatter; however, four-handed dentistry and appropriate use of rubber dam should remain the primary mitigating factors. Further research is required on fine-particle aerosol and air filtration systems to robustly determine the safety of procedures within open settings.

The results need to be interpreted with caution as they cannot be directly extrapolated to aerosolised SARS-CoV-2 spread in a real patient, where saliva, blood, tooth and bone fragments would generate a more complex aerosol, and research in this area of SARS-CoV-2 behaviour is limited. In addition, extrapolation of these results to primary care or hospitals without a HEPA-filtered air exchange system needs caution, as the behaviour and characteristics of air flow are unlikely to be directly comparable. Nevertheless, the results of this study provide a general overview of splatter behaviour and insight into mitigating risk during AGPs for the dental profession.

**Table:** ***Appendix 1*** Air turbine procedure description including procedure detail, operatory type, duration of procedure and scavenging variation. EOS = extraoral scavenging, HVS = high-volume suction, ES = saliva ejector, RD = rubber dam. Frequency (n) denotes the mean number of times sites within that procedure were contaminated and mean intensity (%) of contamination. Clinician and assistant sites are reported separately

Procedure	Code	Operatory	Duration of procedure	Variation of procedure	Overall frequency of operatory site contamination (n)	Overall mean intensity operatory (%)	Maximum intensity operatory (%)	Maximum intensity operatory site name	Overall frequency of clinician contamination (n)	Overall mean intensity clinician (%)	Maximum intensity clinician (%)	Maximum intensity clinician site	Overall frequency of assistant contamination (n)	Overall mean intensity assistant (%)	Maximum intensity assistant (%)	Maximum intensity assistant site
Occlusal cavity preparation of 36; veneer preparation of 31 and 21 using air turbine handpiece	A1	Closed	20 minutes	HVS and SE	10	1.5	62.64	Patient	7	4.24	51.94	Visor front	2.00	0.22	3.42	Visor front
	A2			EOS, HVS and SE	10	0.61	10.49	Patient	6	2.31	19.11	Left shoe	2.00	0.04	0.53	Left shoe
	A3	Open		HVS and SE	11	9.49	94.12	Patient	6	0.22	1.47	Visor front	4.00	3.20	44.24	Visor front
	A4			EOS, HVS and SE	6	1.7	5.24	Patient	4	0.30	3.55	Visor front	4.00	0.36	4.20	Left forearm
	A5			HVS and SE (short visor)	7	15.87	98.88	Patient	5	0.31	1.82	Chest	6.00	0.39	1.82	Chest
	A6			EOS, HVS and SE (short visor)	6	1.33	3.58	Patient	7	0.40	2.08	Visor front	5.00	0.17	1.67	Right forearm
Labial veneer and palatal access cavity preparation of 21 using air turbine handpiece	B1	Closed	10 minutes	RD, HVS and SE	9	0.34	6.46	Patient	5	1.57	11.48	Chest	3.00	0.15	1.45	Head back
	B2			RD, EOS, HVS and SE	7	0.11	0.99	Site 18	6	0.82	5.49	Visor left	2.00	0.06	0.62	Left shoe
Occlusal cavity preparation of 36 with air turbine handpiece	C1	Closed	5 minutes	HVS	8	3.28	88.8	Patient	6	0.80	4.80	Chest	4.00	0.60	7.01	Right forearm
	C2			EOS and HVS	6	1.91	69.01	Site 24	6	1.38	13.17	Chest	3.00	0.28	3.80	Left forearm
	C3			EOS and SE	4	0.19	4.72	Patient	1	0.06	1.12	Mask	1.00	0.13	2.29	Left forearm
	C4			No suction	10	2.53	56.93	Patient	7	3.24	23.28	Chest	No assistant present			
Occlusal cavity preparation of 46 with air turbine handpiece	D1	Open	5 minutes	HVS and SE	3	1.27	2.87	Patient	4	0.12	0.84	Right forearm	3.00	0.11	0.72	Visor front
	D2	Closed		RD, HVS and SE	2	0.03	0.83	Site 18	5	0.34	2.11	Chest	3.00	0.17	2.04	Right forearm
	D3			RD, EOS, HVS and SE	4	0.18	4.29	Patient	4	0.19	1.04	Left forearm	3.00	0.13	0.95	Right forearm

**Table:** ***Appendix 2*** Ultrasonic scaler and triple air procedure description including procedure detail, operatory type, duration of procedure and scavenger variation. EOS = extraoral scavenging, HVS = high-volume suction, ES = saliva ejector, RD = rubber dam. Frequency (n) denotes the mean number of times sites within that procedure were contaminated and mean intensity (%) of contamination. Clinician and assistant sites are reported separately

Procedure	Code	Operatory type	Duration of procedure	Variation of procedure	Overall frequency of operatory site contamination (n)	Overall mean intensity operatory (%)	Maximum intensity operatory (%)	Maximum intensity operatory site name	Overall frequency of clinician contamination (n)	Overall mean intensity clinician (%)	Maximum intensity clinician (%)	Maximum intensity clinician site	Overall frequency of assistant contamination (n)	Overall mean intensity assistant (%)	Maximum intensity assistant (%)	Maximum intensity assistant site
Full mouth debridement using ultrasonic scaler	E1	Closed	7 minutes	70% speed, HVS and SE	0	0.00	0.00	None	2	0.07	0.73	Chest	1	0.03	0.57	Right shoe
	E2			70% speed, EOS, HVS and SE	4	0.05	1.19	Patient	2	0.05	0.71	Chest	0	0.00	0.00	None
	E3			100% speed, HVS and SE	2	0.32	10.26	Nurse's chair	1	0.10	1.72	Chest	0	0.00	0.00	None
	E4			100% speed, EOS, HVS and SE	2	0.14	5.96	Patient	1	0.05	0.83	Chest	0	0.00	0.00	None
	E5			100% speed, SE	4	1.68	73.59	Patient	3	0.17	1.46	Right forearm	No assistant present			
	E6			100% speed, EOS and SE (no HVS)	1	0.21	9.33	Patient	2	0.28	3.76	Right shoe	No assistant present			
	E7	Open		100% speed, HVS and SE	3	0.02	1.26	Patient	2	0.07	0.79	Head top	0	0.00	0.00	None
	E8			100% speed, EOS and SE (no HVS)	1	0.01	0.73	Site 71	4	0.22	1.86	Left shoe	No assistant present			
Triple air syringe (both air and water at maximum pressure) into 36 occlusal cavity	F1	Closed	5 minutes continuously	HVS	5	0.16	3.19	Bracket table	4	0.47	3.78	Right shoe	2	0.05	0.64	Visor front
	F2			EOS, HVS and SE	4	0.05	0.77	Site 25	3	0.14	1.30	Head back	4	0.24	1.65	Right forearm

**Table:** ***Appendix 3*** Surgical procedure description including procedure detail, operatory type, duration of procedure and scavenger variation. EOS = extraoral scavenging, HVS = high-volume suction, ES = saliva ejector, RD = rubber dam. Frequency (n) denotes the mean number of times sites within that procedure were contaminated and mean intensity (%) of contamination. Clinician and assistant sites are reported separately

Procedure	Code	Operatory type	Duration of procedure	Variation of procedure	Overall frequency of operatory site contamination (n)	Overall mean intensity operatory (%)	Maximum intensity operatory (%)	Maximum intensity operatory site name	Overall frequency of clinician contamination (n)	Overall mean intensity clinician (%)	Maximum intensity clinician (%)	Maximum intensity clinician site	Overall frequency of assistant contamination (n)	Overall mean intensity assistant (%)	Maximum intensity assistant (%)	Maximum intensity assistant site
Surgical sectioning of 37 and 38	G1	Closed	10 minutes	Surgical suction and SE	1	2.14	96.45	Patient	1	0.15	2.74	Chest	3	5.44	45.23	Chest
	G2			Surgical suction	1	1.3	58.71	Patient	1	1.36	24.52	Chest	4	7.30	71.07	Chest
	G3			EOS and surgical suction	1	1.66	74.7	Patient	2	0.28	4.10	Visor right	3	1.63	21.80	Chest
Surgical sectioning of 47 and 48	H1	Closed	10 minutes	Surgical suction and SE	1	2.01	90.24	Patient	2	0.09	1.13	Chest	5	3.51	53.13	Chest
	H2			Surgical suction only	1	0.77	34.71	Patient	2	0.08	1.13	Visor front	6	3.26	56.15	Chest
Implant osteotomy preparation of 36	I1	Closed	Sequential use of implant preparation drills 2.2 mm, 2.8 mm and 3.5 mm	Surgical suction	1	0.04	1.72	Patient	0	0.00	0.00	None	3	0.50	4.17	Visor front
Implant osteotomy preparation of 46	J1	Closed	Sequential use of implant preparation drills 2.2 mm, 2.8 mm and 3.5 mm	Surgical suction	0	0	0	None	1	0.04	0.64	Chest	2	0.08	0.91	Chest
	J2			Surgical suction and SE	1	2	90.15	Patient	0	0.00	0.00	None	4	2.77	46.55	Chest
